# Combined ensiling and hydrothermal processing as efficient pretreatment of sugarcane bagasse for 2G bioethanol production

**DOI:** 10.1186/s13068-018-1338-y

**Published:** 2018-12-20

**Authors:** Morten Ambye-Jensen, Riccardo Balzarotti, Sune Tjalfe Thomsen, César Fonseca, Zsófia Kádár

**Affiliations:** 1 Center for BioProcess Engineering, Department of Chemical and Biochemical Engineering, Technical University of Denmark, DTU, Søltofts Plads 229, 2800 Kgs. Lyngby, Denmark; 2 Bioenergy Unit, Laboratório Nacional de Energia e Geologia, I.P., Estrada do Paço do Lumiar 22, 1649-038 Lisbon, Portugal; 3 Present Address: Department of Engineering, Biological and Chemical Engineering, Aarhus University, Finlandsgade 22, 8200 Aarhus N, Denmark; 4 Present Address: Department of Geosciences and Natural Resource Management (IGN), University of Copenhagen, Rolighedsvej 23, 1958 Frederiksberg C, Denmark; 5 Present Address: Section for Sustainable Biotechnology, Department of Chemistry and Bioscience, Aalborg University, A C Mæyers Vænge 15, 2450 Copenhagen SV, Denmark

**Keywords:** Sugarcane bagasse, Combined pretreatment, Ensiling, Hydrothermal pretreatment, Enzymatic hydrolysis, Ethanol fermentation

## Abstract

**Background:**

Ensiling cannot be utilized as a stand-alone pretreatment for sugar-based biorefinery processes but, in combination with hydrothermal processing, it can enhance pretreatment while ensuring a stable long-term storage option for abundant but moist biomass. The effectiveness of combining ensiling with hydrothermal pretreatment depends on biomass nature, pretreatment, and silage conditions.

**Results:**

In the present study, the efficiency of the combined pretreatment was assessed by enzymatic hydrolysis and ethanol fermentation, and it was demonstrated that ensiling of sugarcane bagasse produces organic acids that can partly degrade biomass structure when in combination with hydrothermal treatment, with the consequent improvement of the enzymatic hydrolysis of cellulose and of the overall 2G bioethanol process efficiency. The optimal pretreatment conditions found in this study were those using ensiling and/or hydrothermal pretreatment at 190 °C for 10 min as this yielded the highest overall glucose recovery yield and ethanol yield from the raw material (0.28–0.30 g/g and 0.14 g/g, respectively).

**Conclusion:**

Ensiling prior to hydrothermal pretreatment offers a controlled solution for wet storage and long-term preservation for sugarcane bagasse, thus avoiding the need for drying. This preservation method combined with long-term storage practice can be an attractive option for integrated 1G/2G bioethanol plants, as it does not require large capital investments or energy inputs and leads to comparable or higher overall sugar recovery and ethanol yields.

## Introduction

Sugarcane bagasse (SCB) is an abundant lignocellulosic agro-industrial by-product generated during sugar manufacturing, after the sugar juice is extracted from the sugarcane crop. The annual sugarcane production was around 1900 million tones worldwide in 2014 [1]. Brazil, China, India, and Thailand are the main sugarcane producers, all together representing 70% of the total worldwide sugarcane production, with Brazil alone having a 40% share. One ton of harvested sugarcane generates approximately 275 kg of bagasse with 50% moisture content [2]. Most of the bagasse is burned for electricity production to supply the energy needed in the mills [3], but combustion to electricity is often inefficient. Alternatively, sugarcane bagasse, which represents an enormous amount of waste material, can be used for second-generation (2G) bioethanol production.

Having one of the most advanced biofuel programs significantly based on sugarcane ethanol, Brazil has a great interest in the integration of novel and more efficient technologies for the upgrading of the lignocellulosic biomass obtained in the well-establish first-generation (1G) process. The development of 2G bioethanol processes, especially in combination with existing 1G bioethanol industries, is one attractive option, as it can utilize existing energy supply systems and equipment, such as bioreactors for fermentation and distillation columns. Besides, it is advantageous that sugarcane bagasse is already available on-site, thus decreasing logistics costs. Moreover, since only about one-third to half of the available bagasse is required to produce the energy needed for the 1G bioethanol process, the integration of the 2G process is estimated to increase the total ethanol production by 40% [4]. The advantages of the integrated 1G/2G bioethanol production were already emphasized [5]. Furlan et al. [6] compared the economic feasibility of 1G, 1G plus electric energy, and integrated 1G/2G bioethanol biorefinery plants in Brazil and concluded that the dedicated 1G/2G bioethanol biorefinery was most advantageous. Furthermore, different levels of integration between 1G and 2G ethanol production showed better economic results compared to stand-alone plants, especially when advanced hydrolysis techniques and pentose fermentation were included (5). The usage of the whole sugarcane lignocellulosic biomass (bagasse, straw and tops) can significantly increase 2G ethanol production when compared to the use of the bagasse alone [7]. It should be noted that 1G bioethanol plants typically run their processes for only 8–10 months per year, since sugarcane could not be harvested in the rainy season. Therefore, technologies allowing long-term storage of the bagasse are an attractive option for integration of 1G/2G bioethanol biorefineries. The utilization of other crops during the off-season period is an alternative, but it requires further logistics and process adaptations as in the case of corn-starch bioethanol production [8].

Pretreatment is one of the most important and expensive steps in lignocellulosic ethanol biorefineries. For the utilization of sugarcane bagasse, the costs of alkaline pretreatment and the long process time of biological pretreatment have been found to be the limiting factors for large-scale 2G bioethanol applications [9]. Hydrothermal processing has been considered a cost-effective and environmentally friendly pretreatment method as it (i) does not require the addition and recovery of chemicals; (ii) has limited equipment corrosion problems; (iii) has simple and economical operation [10].

Sugarcane bagasse has a dry matter content of approximately 50% which is almost the optimal value for hydrothermal pretreatment. Therefore, dry biomass storage would not be necessary and advantageous, as drying would not only increase costs but would also potentially increase the recalcitrance of the biomass [11]. On the other hand, due to its natural moisture content, sugarcane bagasse during storage can be easily subjected to spontaneous deterioration by different microorganisms and significant reduction of available saccharides.

Ensiling provides a controlled solution for wet storage and long-term preservation of the material, as fermentation with lactic acid bacteria prevents extensive biomass degradation by other microbes. Due to acidification by organic acids during ensiling, the decreased pH prevents the growth and proliferation of degrading microorganisms (e.g., fungi and competing bacteria able to degrade polysaccharides), and preserves the biomass. During storage, the low pH can initiate a pretreatment effect on the biomass by partly degrading the biomass structure [12]. This preservation method allows for a biomass pretreatment combined with storage practice, which does not require large capital investments or energy inputs.

Ensiling has been previously combined with hydrothermal pretreatment (HTT) on wheat straw and grass, with stream explosion on hemp, and in combination with fungal delignification on wheat straw [12–15]. Combination of two biological pretreatments (ensiling and fungal pretreatment) can be advantageous in developing countries, where smaller bioenergy production units are favorable due to limited biomass logistics [15]. Combination of ensiling with steam pretreatment did not show significant effect for the conversion of hemp to ethanol [14]. However, ensiling of wheat straw prior to HTT has shown significant improvement of pretreatment and overall 2G bioethanol process, being proposed to reduce pretreatment costs on large-scale operations by reducing the HTT temperature or by increasing overall conversion yields [12]. Similar experiments on grass have shown that the improvements are significantly lower than on wheat straw, which emphasizes that the effect of ensiling in HTT is biomass dependent [13].

The aim of this study is to analyze the effect of ensiling prior to HTT as an effective combinatorial pretreatment to process sugarcane bagasse for 2G bioethanol production. The organic acids production at low dry matter content during ensiling can catalyze hydrothermal pretreatment, reducing the pretreatment temperature and thus overall energy consumption. The hypothesis is that the conversion of sugarcane bagasse to ethanol similarly to wheat straw will decrease the necessary temperature of HTT if ensiling is used prior. The pretreatment effectiveness was measured by glucose recovery after enzymatic hydrolysis and by subsequent bioethanol production by fermentation.

## Results and discussions

### Ensiling of sugarcane bagasse

After 4 weeks ensiling of SCB minimal weight loss (0.1% w/w) was observed (Table 1) indicating that the biomass was well preserved. This result indicates an efficient ensiling process, a consequence of the anaerobic conditions applied and of the prevalence of the lactic acid bacteria inoculated.

**Table 1:** Dry matter loss, pH, and the most significant organic compounds in water extractives in ESCB after 4 weeks of ensiling (% w/w of DM)

DM loss (% w/w)	0.1
pH	4.1
Glucose	0.4 ± 0.00
Xylose	3.6 ± 0.02
Lactic acid	4.5 ± 0.02
Acetic acid	3.5 ± 0.01
Total*	8.0

* Total includes the fatty acids

The produced lactic and acetic acids, 4.5 and 3.5% (w/w), respectively, caused a pH drop from the initial 7.0 to 4.1. From the 7% (w/w) xylose supplemented for the ensiling process, approximately half (3.6% w/w) was recovered in the water extract from ESCB. Using the same approach for ensiling of wheat straw, Ambye-Jensen et al. [12] recovered only 1.3% (w/w) xylose at the end of the process. Considering the stoichiometry of xylose fermentation to lactic and acetic acids, the 3.4% (w/w) xylose consumed would yield at maximum 2.0 and 1.4% (w/w) lactic and acetic acids, respectively. The high concentration of organic acids obtained in the ensiling process suggests that the supplemented xylose is not the only carbon source used in the anaerobic fermentation process. Dewar et al. [17] showed that the production of organic acids in biomass silage led to partial hydrolysis of the hemicellulose fraction. However, in the case of wheat straw ensiling, the released xylose from hemicellulose counted for a negligible fraction [12]. In the present study, the maintenance of the hemicellulose content in the ESCB (Table 2) implies that the contribution from hemicellulose for the acid products obtained during ensiling can mainly result from deacetylation of this fraction. The lower amount of glucan and water extractives in ESCB when compared to SCB (Table 2) indicates that some carbohydrates have been utilized by the bacteria during ensiling. Taking into account that SCB contains residual non-structural carbohydrates from sugarcane processing (e.g., sucrose), the use of xylose supplementation to boost ensiling may be avoided.

**Table 2:** Solid fiber yields after HTT and chemical composition of the solid fibers from raw sugarcane bagasse (SCB), hydrothermal pretreated sugarcane bagasse (HTT SCB), ensiled sugarcane bagasse (ESCB), and hydrothermal pretreated ensiled sugarcane bagasse (HTT ESCB)

	Cellulose (w/w% of DM)	Hemicellulose (w/w% of DM)	Lignin (w/w% of DM)	Extractives^a^ (w/w% of DM)	Solid fiber yield (%)
SCB	36.1 ± 4.2	22.3 ± 2.1	16.9 ± 0.6	18.6 ± 0.0	
HTT SCB 160 °C	41.8	23.8	21.8		85.5
HTT SCB 170 °C	43.3	22.0	22.2		78.4
HTT SCB 180 °C	47.0 ± 1.7	19.1 ± 0.6	23.1 ± 0.2		80.8 ± 0.03
HTT SCB 190 °C	49.4	13.6	24.9		68.1
ESCB	32.5 ± 1.3	22.3 ± 1.2	18.7 ± 0.1	13.0 ± 0.9	
HTT ESCB 160 °C	40.9	23.9	21.2		75.5
HTT ESCB 170 °C	44.3	19.8	22.8		71.9
HTT ESCB 180 °C	45.1 ± 2.4	17.3 ± 0.1	23.7 ± 0.1		72.6 ± 0.03
HTT ESCB 190 °C	48.6	11.9	25.4		63.8

^a^Extractives are the result of water extraction

### Effect of ensiling and hydrothermal pretreatment in the composition of solid fibers

Hydrothermal pretreatment was carried out on both SCB and ESCB. The slurry after pretreatment was separated into solid and liquid fractions. The composition of the solid fibers from untreated SCB (SCB) and hydrothermally pretreated SCB (HTT SCB) was compared to those from ensiled SCB (ESCB) and hydrothermally pretreated ESCB (HTT ESCB) (Table 2).

The comparison of the glucan content of SCB with that of ESCB shows that ensiling preserves most of the cellulose (90%). Still, this result contrasts with that of wheat straw ensiling, where almost all the cellulose (99%) was preserved [12]. During HTT at high temperatures (160–190 °C), water acts as a weak acid and initiates the mechanism of autohydrolysis and acetyl groups are released from hemicellulose, further enabling the solubilization of this fraction [10]. This feature can be observed from the results in Table 2 comparing untreated and pretreated samples. Since hemicellulose was partially solubilized with HTT, cellulose and lignin were up-concentrated in the solid fibers of pretreated biomass, a phenomenon that is intensified at higher temperatures. However, no significant impact of ensiling was observed in the cellulose and lignin content of the solid fibers. Cellulose obtained from HTT SCB and HTT ESCB between 160 and 190 °C ranged between 41.8 and 49.4% in HTT SCB, and between 40.9 and 48.6% in HTT ESCB, representing a glucan recovery of more than 90% in relation to the SCB and ESCB content in all the HTT conditions tested (Table 2, see next section). The increase of temperature in HTT also up-concentrated lignin in the solid fibers, both on SCB and ESCB, ranging from 21 to 22% at 160 °C to around 25% at 190 °C. During hydrothermal pretreatment, lignin is melted, diffused out of the cell wall matrix, and redistributed on the fibers surface, thus HTT rearranges the lignin fraction of the biomass rather than removing it [18].

The combination of ensiling with HTT impacted differently in the composition of the solid fibers, mainly in the hemicellulose content. At 170 °C, ensiling extended the overall effect of the pretreatment reducing hemicellulose content from 22.0% (HTT SCB 170 °C) to 19.8% (HTT ESCB 170 °C). In fact, the solubilization of hemicellulose at 170 °C after ensiling (HTT ESCB 170 °C) is similar to the one obtained at 180 °C without prior ensiling (HTT SCB 180 °C)—hemicellulose content of solid fibers of 19.8 and 19.1%, respectively. A more extended hemicellulose solubilization was obtained with HTT at 190 °C, where the hemicellulose in the solid fibers was nearly halved for both SCB (from 22.3 to 13.6%) and ESCB (from 22.3 to 11.9%). The impact of ensiling on hemicellulose solubilization after HTT can be observed comparing HTT ESCB and HTT SCB at 170, 180, and 190 °C. HTT treatment at 160 °C appears to be too mild to observe an effect of ensiling with virtually no solubilization of hemicellulose in both HTT SCB and HTT ESCB.

### Mass balance after pretreatment

A complete mass balance was formulated after hydrothermal treatment since the liquid fraction obtained was also characterized. The cellulose and hemicellulose in the solid fraction and the respective carbohydrates obtained in the liquid fraction were used to calculate the recovery factors.

Virtually, all the cellulose in the solid fibers were preserved in all cases after hydrothermal pretreatment of both SCB and ESCB, with a recovery factor above 90%.

**Fig. 1:**
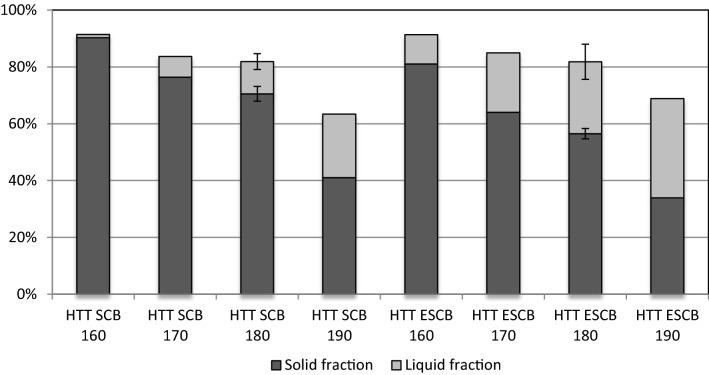
Recovery of hemicellulose (xylan) in solid and liquid fractions after HTT SCB and HTT ESCB at different temperatures

As earlier described, HTT solubilizes hemicellulose but the high temperatures can lead to significant degradation of the solubilized carbohydrates [19]. Figure 1 represents the hemicellulose (xylose) recovery factors both in solid and in liquid fractions. The increased temperature of HTT increased the solubilization of hemicellulose both on SCB and ESCB. Total recovery varies from the 90% of HTT at 160 °C to the 60–70% (SCB–ESCB) for HTT at 190 °C (Table 2). In most of the cases (except 190 °C), hemicellulose was mainly (> 50%) recovered in the solid fraction.

In general, the solubilization of hemicellulose was higher when ensiling was applied before HTT, as a decrease of hemicellulose content in the solid fraction was observed at the same temperatures. For example, at 170 °C, the recovery factors in solid and liquid fractions were, respectively, 76% and 7% for SCB and 64% and 21% for ESCB, while at 180 °C reached 70.5% and 11.3% for SCB and 56.5% and 25.3% for ESCB (Fig. 1). This supports the hypothesis that ensiling has effect on HTT severity in terms of hemicellulose solubilization [12], with the additional acid produced during ensiling functioning as catalyst during HTT. The lower recovery factors at higher temperatures most probably result from carbohydrate (xylose) degradation into furan derivatives (e.g., furfural) [19].

### Enzymatic hydrolysis of pretreated SCB solid fibers

Enzymatic hydrolysis of the untreated and hydrothermal pretreated (HTT) solid fibers of SCB and ESCB was performed in order to evaluate the pretreatment efficiency. The increased cellulose accessibility during HTT is due to the partial hydrolysis of hemicellulose hydrolysis and relocation of lignin. Commercial cellulases and hemicellulases were applied for the enzymatic hydrolysis of the solid fibers. Table 3 shows the glucose and xylose recovery after enzymatic hydrolysis.

**Table 3:** Glucose and xylose recovery after enzymatic hydrolysis of raw sugarcane bagasse (SCB), of ensiled sugarcane bagasse (ESCB), or of the solid fraction from hydrothermal pretreated sugarcane bagasse (HTT SCB) or hydrothermal pretreated ensiled sugarcane bagasse (HTT ESCB)

	Glucose recovery yield (enzymatic hydrolysis)%^a^	Overall glucose recovery yield (g of glucose/g of raw material)^b^	Xylose recovery yield (enzymatic hydrolysis)%^a^	Overall xylose recovery yield (g of xylose/g of raw material)^b^
SCB	18.4 ± 1.0	0.07	9.9 ± 0.7	0.02
HTT SCB 160 °C	30.5 ± 0.8	0.12	25.5 ± 1.1	0.06
HTT SCB 170 °C	46.3 ± 6.0	0.17	49.6 ± 4.6	0.09
HTT SCB 180 °C	60.4 ± 4.7	0.25	74.1 ± 4.1	0.12
HTT SCB 190 °C	75.5 ± 1.5	0.28	95.1 ± 0.8	0.10
ESCB	25.5 ± 0.9	0.09	18.1 ± 0.8	0.04
HTT ESCB 160 °C	31.7 ± 4.0	0.11	31.5 ± 4.5	0.06
HTT ESCB 170 °C	50.8 ± 0.8	0.18	64.6 ± 2.7	0.10
HTT ESCB 180 °C	68.6 ± 5.2	0.24	84.9 ± 5.0	0.11
HTT ESCB 190 °C	86.9 ± 3.5	0.30	95.0 ± 3.3	0.08

^a^Glucose or xylose recovery yield expressed as glucose or xylose recovered in % of glucose or xylose equivalents in the solid fibers used in the enzymatic hydrolysis process

^b^Overall glucose or xylose recovery yield is the glucose or xylose obtained from the raw material after pretreatment (if used) and enzymatic hydrolysis, expressed in g of glucose or xylose per g of raw material

The cellulose conversion increased with the HTT temperature, as denoted by the higher glucose recovery yields, which reaches more than 85% in the HTT ESCB 190 °C (Table 3). The highest glucose recovery without ensiling was 75.5%, at the highest HTT temperature (Table 3). Lower HTT temperatures resulted in significantly lower cellulose conversion: for example, at 170 °C the efficiency of enzymatic hydrolysis was only 61% of that at 190 °C. The low cellulose digestibility (< 50%) at 160 and 170 °C showed that the severity at these temperatures was not sufficient enough to disrupt the lignocellulose structure both with and without means of ensiling. The best results obtained for enzymatic hydrolysis of HTT SCB were similar or higher than the previously reported. Wang et al. [20] reported 68.3% enzymatic hydrolysis yield of SCB with liquid hot water pretreatment at 180 °C for 20 min when a fed-batch process was applied. In turn, Silva et al. [21] and Rocha et al. [22] achieved only 56.9% hydrolysis yield at 190 °C for 10 min, compared to our results which reached the 70.1% under similar severity conditions. This can be most likely due to the (i) difference in biomass composition as in our study, the sugarcane bagasse had significantly lower lignin content or (ii) the applied higher solid loading (10%) at Silva et al. [21] and Rocha et al. [22]. On untreated SCB, those authors achieved a hydrolysis yield of only 6.0%, while in this work, cellulose conversion yield of SCB achieved 18.4%. Those authors were able to increase significantly the glucose recovery yield when delignification was applied after HTT. When HTT at 190 °C for 10 min was followed by delignification (with NaOH 1.0% (w/v), 100 °C, 1 h), the enzymatic hydrolysis yield increased to 89.2% which is similar to that achieved in this work when HTT was combined with ensiling (86.9%).

The data also showed that even though ensiling improved the cellulose digestibility when combined with HTT, ensiling as a stand-alone pretreatment was not effective, as only 23.0% (0.09 g/g) of the available glucose in the raw material could be obtained. In fact, the glucose recovery yields after enzymatic hydrolysis were improved when ensiling was combined with HTT. At all temperatures, the combination of ensiling and HTT led to an increase in cellulose conversion of 10–15% in relation to stand-alone HTT. The highest overall glucose recovery yield (0.30 g/g) was achieved with HTT ESCB at 190 °C, which represents approx. 75% of glucose equivalents in the raw material, 5% more than with HTT SCB at the same temperature. The positive effect of ensiling combined with HTT can be both due to the long impregnation time with organic acids during ensiling, and due to the contribution of lower pH at the beginning of HTT, which increases HTT severity [12].

Similar experiments were performed on wheat straw (WS) [12]. The cellulose digestibility of the HTT WS increased with the HTT temperature similarly to SCB, especially from 180 to 190 °C, where the glucose recovery yield raised from 45.9 to 71.5%. The prior ensiling before HTT of WS resulted in an increase of the glucose recovery yield up to 78.7% and 73.5%, respectively, for 180 °C to 190 °C. The benefits of wheat straw ensiling combined with HTT pretreatment on higher cellulose conversion were observed only at lower (170 °C and 180 °C) HTT temperatures. The findings potentially enable a considerable decrease in the necessary process temperature in HTT of WS, thereby having a positive effect on large-scale pretreatment costs. On SCB, an increased glucose recovery was observed at all temperatures when ensiling was applied prior to HTT. In this case, the recommendation is, thus, not to decrease HTT temperature as with WS. The benefits, found in this study, of ensiling SCB prior to HTT rely in the higher overall glucose recovery yield when compared with that obtained without ensiling at high HTT temperature. Moreover, the ensiling process will prevent fast deterioration of SCB in a combined 1G/2G ethanol plant.

Both with WS and SCB, ensiling was found to provide significant advantages when combined with HTT, but these benefits largely depend on the type of biomass. While ensiling of WS in combination with HTT facilitated a reduction in HTT temperature up to 20 °C, ensiling of SCB consistently increased HTT efficiency in terms of cellulose digestibility in 10–15%. The impact of structural differences of biomass has been further confirmed when similar experiments were performed on grass [13]. Even though the ensiling of grass prior HTT caused also increased solubilization and higher concentration of cellulose in the solid fibers compared to non-ensiled grass, the improvement was significantly lower than that found on WS and now on SCB.

On WS, it was concluded that substantial more released sugar was gained than the 7% xylose was used to facilitate the ensiling process. In contrast, with grass, the loss of water-soluble carbohydrates during ensiling had a large drawback on the overall sugar recovered. The positive effect of ensiling SCB prior HTT can be quantified by comparing the overall sugar recovery yields at the same temperatures. Comparing the released glucose at 190 °C from SCB and ESCB, it can be concluded that more released sugar was achieved than was added to facilitate ensiling. Furthermore, the results of excess xylose in the water extract of ESCB suggest that supplementation of sugars is not needed to boost ensiling in the case of SCB.

Hemp silage was ensiled without any additives and the results indicated that the silage was well preserved [14]. However, steam explosion pretreatment in combination with ensiling could not increase the overall glucose recovery yield.

In other studies, pretreatment of sugarcane bagasse by steam explosion without addition of SO_2_ after one month storage with lactic acid led to 79% glucose recovery yield after enzymatic hydrolysis [23], which was similar to the pretreatment when SO_2_ was only used as the impregnating agent. The results, however, also showed in that study that longer storage time with lactic acid had slightly negative impact. In our study, ensiling was performed for 1 month but the isolated effect of the storage duration was not assessed and should be assessed in future studies.

The digestibility of hemicellulose in the solid fibers is higher at higher HTT temperatures, the same trend observed for cellulose digestibility. The xylose recovery yields after enzymatic hydrolysis were 15–30% higher for the ensiled samples when processed by HTT at lower temperatures (160–180 °C) (Table 3), whereas at HTT 190 °C, the percentage of xylose recovery from the solid fibers was 95% for both ensiled and not ensiled SCB. The overall xylose recovery yield does not increase after 180 °C as the overall glucose does. This may be explained by the significant degradation of the released xylose at the highest HTT temperature tested (190 °C), in line with the lower hemicellulose conversion observed (see Fig. 1).

These results showed that the highest overall sugar recovery is when ensiling is combined with the highest HTT temperature (190 °C). Here, the digestibility of cellulose is the highest and the overall xylose recovery is similar to that of 180 °C, since hemicellulose digestibility is counterbalanced with xylose degradation in the liquid fraction at 190 °C.

### Effect of combining ensiling and hydrothermal treatment on ethanol production by SSF

Enzymatic hydrolysis is a good tool for the evaluation of pretreatment efficiency. However, it does not provide complete picture for the implementation of a 2G bioethanol production process. Therefore, the combination of ensiling with HTT was also evaluated by enzymatic hydrolysis and fermentation of SCB, HTT SCB, ESCB, and HTT ESCB solid fibers. The process was carried out under simultaneous saccharification and fermentation (SSF) with a short pre-hydrolysis using the industrial glucose-fermenting yeast *S. cerevisiae* Ethanol Red^®^, which is unable to ferment xylose. At the end of the SSF process, samples were analyzed to quantify ethanol (Table 4).

**Table 4:** Ethanol production after SSF of raw sugarcane bagasse (SCB), hydrothermally treated sugarcane bagasse (HTT SCB), ensiled sugarcane bagasse (ESCB), and ensiled hydrothermally treated sugarcane bagasse (HTT ESCB) (10% solid fibers)

	Ethanol production (g/l)	SSF ethanol yield %^a^	Overall ethanol yield (g ethanol/g raw material)^b^
SCB	2.7 ± 0.0	14.5	0.03
HTT SCB 160 °C	4.6 ± 0.0	21.7	0.04
HTT SCB 170 °C	7.1 ± 0.0	32.2	0.06
HTT SCB 180 °C	12.6 ± 0.1	52.7	0.11
HTT SCB 190 °C	19.0 ± 0.0	75.3	0.14
ESCB	3.3 ± 0.0	19.9	0.04
HTT ESCB 160 °C	5.3 ± 0.0	25.6	0.04
HTT ESCB 170 °C	10.1 ± 0.0	44.6	0.08
HTT ESCB 180 °C	14.0 ± 0.1	60.8	0.11
HTT ESCB 190 °C	19.7 ± 0.1	79.3	0.14

^a^% of theoretical ethanol yield obtained from the glucose equivalents present in the solid fibers used in the SSF process

^b^Overall ethanol yield is the ethanol obtained from the raw material after pretreatment (if used) and SSF, expressed in g of ethanol per g of raw material

SCB without any pretreatment resulted in only 14.5% ethanol yield of the theoretical. Ensiling as a stand-alone pretreatment method (ESCB) could increase it only up to 19.9% ethanol yield. Ensiling combined with HTT at 160 °C both on SCB and ESCB resulted in very low ethanol yield (< 30%), indicating that the severity of the pretreatment was not efficient as previously observed in dedicated cellulose digestibility tests. These values are, however, still higher than was reported by da Cruz et al. [24], where SCB pretreated at 160 °C for 12.5 min resulted in only 11% ethanol yield.

At HTT 170 °C ensiling improved ethanol yield by a factor of 1.4, boosting ethanol production yield from 32.2% with HTT SCB to 44.6% with HTT ESCB. At higher temperatures, the obtained ethanol yield was still higher on ESCB than SCB, but the factor decreased to 1.2 at 180 °C and was not much improved at 190 °C.

The maximum ethanol concentration found during SSF experiments is the one of HTT ESCB at 190 °C with 19.7 g/l corresponding to almost 80% ethanol yield based of glucose equivalents in the solid fibers.

The obtained ethanol production results in this study are higher than the ones reported by da Cruz et al. [24] where a detailed full factorial design was performed on SCB testing HTT temperature between 160 and 200 °C and the time from 5 to 20 min. The maximum glucose recovery yield was above 80%, while the final ethanol yield was close to 70% of the theoretical maximum when pretreatment was performed at 190 °C for 17 min, or for 12 min but at elevated temperature at 200 °C. At 190 °C HTT, shorter pretreatment time (10 min), similar enzyme dosage, and higher DM content (10%) for the SSF, we here reached higher ethanol yield (75.3%) from non-ensiled SCB. Liquid hot water pretreatment (similar to HTT) of SCB at 180 °C for 20 min resulted in the final ethanol yield of 88.3% already when SSF was followed after a fed-batch hydrolysis [20]. The maximum overall ethanol yield of 0.14 g of ethanol per g of raw material at 190 °C HTT, both with ensiled and non-ensiled SCB, represents around 70% of the maximum ethanol that can be obtained from the glucan fraction of the raw material.

Producing ethanol from the solid fibers after pretreatment is one option. Another, and more advantageous, could be when the whole hydrolysate is used, thus eliminate separations cost after pretreatment, and also allow conversion of all the carbohydrates. It should also be noted that current metabolic engineered yeasts are able to efficiently ferment xylose with glucose [25]. Therefore, further studies need to address the fermentation of the whole slurry in order to study the effect of both organic compounds produced during ensiling, which can be concentrated in the liquid fraction as well as compounds produced during pretreatment with regard to potential inhibition for ethanol fermentation by the yeasts. These inhibitors could especially influence the fermentation of xylose. Sipos et al. [14] performed steam pretreatment on hemp and on ensiled hemp. When the whole slurry was fermented slightly lower, ethanol yield (71.2%) was observed on ensiled hemp when compared with non-ensiled hemp (74.1%). The lower ethanol yield was explained by the presence of acetic acid at a concentration of 6.8 g/l. It is important to emphasize that the authors applied steam explosion at high temperature (210 °C), so probably other compounds (e.g., sugar degradation products) also contributed to the inhibition. Acetic acid and sugar- and lignin-degradation products are among the inhibitory compounds generated from pretreated lignocellulosic biomass [26]. The individual effect of acetic acid at concentrations up to 6 g/l does not significantly impact in ethanol fermentation with *S. cerevisiae* [27] and at a certain concentration, it can help yeast cells overcoming HMF stress [28]. Higher concentrations than 6 g/l have been reported to result in a decrease in the ethanol yield [27]. Ensiling of SCB resulted in an acetic acid production of 52.5 mg/g DM ESCB. Performing HTT pretreatment at high solid loading (25–35%) on ESCB therefore could potentially result in the concentration of acetic acid at a range of 13–18 g/l. Maize silage has been previously tested for ethanol fermentation at similar conditions [29] and 78% of the theoretical ethanol yield was achieved, similarly to our results on ensiled SCB. During that study, the liquid fraction obtained after each pretreatment (185–195 °C) was tested for toxicity against yeast. Inhibition effect was not observed and it even resulted in slightly higher ethanol yield due to the presence of acetic acid at low (< 6 g/l) concentrations. In our case, assuming high solid loading at pretreatment, the acetic acid concentration is higher which might affect ethanol fermentation negatively. However, studies showed that the inhibitory effect of acetic acid can be overcome by (i) different detoxification methods [30], (ii) adaptation of the applied yeast to inhibitors [31, 32], (iii) performing the fermentation in membrane bioreactor [33].

## Conclusions

The combination of ensiling with HTT was evaluated for the conversion of SCB to ethanol. Ensiling prior to HTT catalyzes hydrothermal pretreatment causing increased hemicellulose solubilization and higher concentration of cellulose in the solid fibers compared to non-ensiled SCB. The effect of the pretreatment was determined by enzymatic hydrolysis and SSF of the solid fibers. Ensiling of sugarcane bagasse improved both the enzymatic hydrolysis and ethanol fermentation.

The effect of ensiling in combination with HTT as pretreatment is, however, highly case- and biomass-specific. While when applied to wheat straw this pretreatment process had previously enabled the decreasing of process temperature for HTT in 20 °C, when applied to SCB, ensiling consistently increases HTT efficiency in terms of cellulose digestibility at temperatures between 170 and 190 °C. For the sugarcane bagasse, the ensiling can provide two beneficial effects: (i) preservation of the material for longer storage periods without drying, (ii) increase sugar recovery and final ethanol yield up to 10%, depending on the HTT temperature used. This process can be optimized and tested at pilot scale to be implemented in integrated 1G/2G sugarcane ethanol plants.

## Materials and methods

### Raw material

Sugarcane bagasse (SCB) (*Saccharum officinarum* L.) originated from Louisiana, USA. The material was harvested in 2011, dried in Wyoming, and shipped to Denmark. Dry matter content of the stored SCB was 90–95%.

### Pretreatment process

Combined ensiling and HTT was tested by the conversion of glucose and xylose after subsequent enzymatic hydrolysis and by ethanol yield after fermentation. The combined pretreatment (HTT ESCB) was compared to the conversion in raw sugarcane bagasse (SCB), ensiled sugarcane bagasse (ESCB), and sole HTT pretreated sugarcane bagasse (HTT SCB).

### Ensiling

Ensiling was carried out on chopped SCB (10 cm) adjusted to 35% final dry matter (DM) content. To initiate ensiling of SCB, 7 g xylose per 100 g DM was added as determined to be optimal by Yang et al. [16]. Each batch of ensiling contained 1.3 kg DM SCB. The ensiling was carried out using a vacuum-based plastic bag system [34] and a Variovac EK10 vacuum packaging machine (Variovac Nordic A/S, DK-7100 Vejle, Denmark). The commercially available inoculum LACTISIL CCM (Chr. Hansen A/S, Hørsholm, Denmark) which consists of freeze dried pure heterofermentative *Lactobacillus buchneri* was applied. A suspension of 0.2 g/l water was prepared and added in the amount of 40 ml/kg SCB to reach an initial inoculum size of 8 mg/l. The plastic bags were opened after 4 weeks. Weight loss was measured for calculation of DM loss. After ensiling, 0.5 kg DM of the ensiled SCB was pretreated hydrothermally.

### Hydrothermal pretreatment (HTT)

Hydrothermal pretreatments (HTT) were carried out in the “Mini IBUS” equipment (Technical University of Denmark, Risø campus). Batches of 0.5 kg DM (DM corrected for volatile fatty acid content) were treated at different temperatures (160, 170, 180, and 190 °C) for 10 min. To verify the reproducibility of HTT, the ESCB pretreated at 180 °C was done in triplicate. After HTT, the pretreatment reactor was cooled down below 70 °C thereby avoiding evaporation of acids, and the material was separated by pressing. Each solid fiber fraction and each liquid fraction were analyzed separately. The solid fibers were kept in the freezer and used to evaluate the process efficiency by enzymatic hydrolysis and ethanol fermentation.

### Biomass analysis

Raw sugarcane bagasse (SCB), ensiled sugarcane bagasse (ESCB), hydrothermally pretreated sugarcane bagasse (HTT SCB), and hydrothermally pretreated and ensiled sugarcane bagasse (HTT ESCB) were analyzed for chemical composition by methods based on standard laboratory analytical procedures developed by National Renewable Energy Laboratory (NREL), US [35]. Deviations from these standard procedures are stated in the following sections. The analysis of the solid fiber fraction included ash content determination, water extraction, ethanol extraction, and strong acid hydrolysis for determining structural carbohydrates and lignin. The liquid fraction of the HTT was analyzed by weak acid hydrolysis.

### Dry matter and ash determination

Dry matter (DM) and ash were determined using a standard method [35]. For non-extracted samples, the method was corrected for organic acid content by the method of Huida et al. [36].

### Water extraction

Samples of 15 g DM biomass from freshly disrupted silage bags were extracted in 225 ml MilliQ water with 225 μl of the antibiotic ampicillin (10 mg/ml solution) to prevent microbial activity during extraction. The extraction samples were shaken for 2 h at 25 °C and 150 rpm. Extracts were analyzed for sugars and acids by HPLC as described below.

### Weak acid hydrolysis of hydrolysates

The liquid fraction from HTT was further analyzed by weak acid hydrolysis to quantify the content of soluble oligomeric carbohydrates. Samples of 10 ml from HTT liquid fraction were autoclaved for 10 min at 121 °C with 4 w/w% H_2_SO_4_. Derived sugars were analyzed by HPLC as described below.

### Ethanol extraction

Lipophilic extraction was carried out by Soxhlet extraction in a reflux condenser for 6 h with 99 w/w% ethanol on water extracted samples of SCB. The amount of ethanol extractives, including volatiles, was defined as the mass of material lost through extraction.

### Determination of structural carbohydrates and lignin

Strong acid hydrolysis was used to measure the carbohydrate and lignin content of the extracted samples, and pretreated biomass, based on the NREL standard laboratory analytical procedure [35].

### Enzymatic hydrolysis

The enzymatic convertibility assay based on commercial Cellic^®^ CTec2 (blend of cellulases) and Cellic^®^ HTec2 (blend of hemicellulases) (Novozymes A/S, Denmark) was used to determine the efficiency of the pretreatment process. Enzymatic hydrolysis of pretreated solids was performed at 5% DM content in a total volume of 25 ml using citrate buffer (50 mM, pH 5) and 0.25 ml sodium azide (2%) at 50 °C, 150 rpm, for 72 h. The applied enzyme loading was 15 FPU/g DM solids of Cellic^®^ CTec2 supplemented with Cellic^®^ HTec2 (9:1 ratio, based on protein content). The enzymatic hydrolysis was performed in triplicates and enzyme blanks were included. Samples were analyzed for carbohydrates by HPLC. Cellulose convertibility was calculated as glucose equivalents recovered from the original cellulose content.

### Ethanol fermentation

Simultaneous saccharification and fermentation (SSF) of raw bagasse (SCB), ensiled bagasse (ESCB), hydrothermal pretreated raw bagasse (HTT SCB), and hydrothermal pretreated ensiled bagasse (HTT ESCB) were performed in 250-ml blue capped flasks containing 10% DM insoluble solid in a total volume of 50 ml. Each flask contained the substrate, citrate buffer (50 mM, pH 5), and enzyme loading at 15 FPU/g DM solids of Cellic^®^ CTec2 supplemented with Cellic^®^ HTec2 (9:1 ratio, based on protein content). A pre-hydrolysis step was performed for 6 h at 50 °C, 140 rpm. After the liquefaction step, the flasks were cooled down and inoculated with 0.1 g cell dry weight of commercial yeast (*Saccharomyces cerevisiae* Ethanol Red^®^, Fermentis). The yeast cells were harvested from an overnight culture grown at 35 °C, 140 rpm, in a 250-ml shake flask containing 50 ml of medium (2.5 g/l (NH_4_)_2_SO_4_, 1 g/l KH_2_PO_4_, 0.3 g/l MgSO_4_.7H_2_O, 2 g/l yeast extract and 50 g/l glucose), and washed twice with demineralized water. After inoculation, the flasks were closed with yeast lockers and incubated at 35 °C, 140 rpm, for 7 days. SSF was performed in triplicates for each sample of solid fiber. Additionally enzyme and substrate blanks were also included. The fermentation was followed by weight-loss measurement. At the end of the incubation time, samples were centrifuged for 5 min at 3500 rpm and the supernatants were analyzed for ethanol by HPLC as described below.

### Analytical method

Carbohydrates (d-glucose, d-xylose, l-arabinose), organic acids (lactic, acetic), and ethanol were quantified by HPLC system equipped with a refractive index detector, a Biorad HPX-87H column (Hercules, CA; USA) at 63 °C, and a mobile phase of 4 mM H_2_SO_4_ at 0.6 ml/min flow rate.

## Abbreviations

HTT: hydrothermal treatment
SCB: sugarcane bagasse
ESCB: ensiled sugarcane bagasse
RSCB: raw sugarcane bagasse
DM: dry matter
HPLC: high-performance liquid chromatography
